# Multimodal data deep learning method for predicting symptomatic pneumonitis caused by lung cancer radiotherapy combined with immunotherapy

**DOI:** 10.3389/fimmu.2024.1492399

**Published:** 2025-01-08

**Authors:** Mingyu Yang, Jianli Ma, Chengcheng Zhang, Liming Zhang, Jianyu Xu, Shilong Liu, Jian Li, Jiabin Han, Songliu Hu

**Affiliations:** ^1^ Harbin Medical University Cancer Hospital, Harbin, China; ^2^ Harbin Institute of Technology, Harbin, China

**Keywords:** lung cancer, radiotherapy, immunotherapy, pulmonary toxicity, radiomics, deep learning

## Abstract

**Objectives:**

The pairing of immunotherapy and radiotherapy in the treatment of locally advanced nonsmall cell lung cancer (NSCLC) has shown promise. By combining radiotherapy with immunotherapy, the synergistic effects of these modalities not only bolster antitumor efficacy but also exacerbate lung injury. Consequently, developing a model capable of accurately predicting radiotherapy- and immunotherapy-related pneumonitis in lung cancer patients is a pressing need. Depth image features extracted from deep learning, combined with radiomics and clinical characteristics, were used to create a deep learning model. This model was developed to forecast symptomatic pneumonitis (SP) (≥Grade 2) in lung cancer patients undergoing thoracic radiotherapy in combination with immunotherapy.

**Methods:**

The prediction was based on CT scans taken prior to the start of thoracic radiotherapy. Retrospective collection of clinical data was conducted on 261 lung cancer patients undergoing a combination of thoracic radiotherapy and immunotherapy from January 2018 to May 2023. Imaging data in the form of pre-RT-CT scans were obtained for all individuals included in the study. The region of interest (ROI) in the lung parenchyma was outlined separately from the tumor volume, and standard radiomic features were obtained through the use of 3D Slicer software. In addition, the images were cropped to a uniform size of 224x224 pixels. Data augmentation techniques, including random horizontal flipping, were employed. The normalized image data was then input into a pre-trained deep residual network, ResNet34, which utilized convolutional layers and global average pooling layers for deep feature extraction. A five-fold cross-validation approach was implemented to construct the model, automatically splitting the dataset into training and validation sets at an 8:2 ratio. This process was repeated five times, and the results from these iterations were aggregated to compute the average values of performance metrics, thereby assessing the overall performance and stability of the model.

**Results:**

The multimodal fusion model developed in this research, which incorporated depth image characteristics, radiomics properties, and clinical data, demonstrated an AUC of 0.922 (95% CI: 0.902-0.945, P value < 0.001). This amalgamated model surpassed the performance of the radiomic feature model (AUC 0.811, 95% CI: 0.786-0.832, P value < 0.001), the clinical information model (AUC 0.711, 95% CI: 0.682-0.753, P value < 0.001), as well as the model that integrated omics attributes with clinical data (AUC 0.872, 95% CI: 0.845-0.896, P value < 0.001) utilizing deep neural networks (DNNs). Comparatively, the radiomic feature model based on random forest (RF) yielded an AUC of 0.576, with a 95% confidence interval of 0.523-0.628. The clinical information model based on RF had an AUC of 0.525, with a 95% confidence interval of 0.479-0.572. When both radiomic features and clinical information were combined in a model based on RF, the AUC improved slightly to 0.611, with a 95% confidence interval of 0.566-0.652.

**Conclusions:**

In this study, a deep neural network-based multimodal fusion model improved the prediction performance compared to traditional radiomics. The model accurately predicted Grade 2 or higher SP in lung cancer patients undergoing radiotherapy combined with immunotherapy.

## 1 Introduction

Lung cancer is a leading contributor to cancer-related deaths worldwide. Immunotherapy has become a revolutionary strategy for transforming the management of lung cancer. Radiotherapy serves as a vital local treatment modality within the comprehensive lung cancer treatment paradigm (1). The integration of immunotherapy with radiotherapy has become the established treatment protocol for locally advanced, inoperable NSCLC. Specifically, results from the PACIFIC trial have shown a remarkable enhancement in 5-year overall survival (OS) and progression-free survival (PFS) percentages in individuals with stage III NSCLC who received durvalumab post simultaneous chemoradiotherapy, exceeding results seen in the control group (2). Consequently, immune consolidation therapy followed by concurrent chemoradiotherapy is currently the established treatment protocol for LA-NSCLC. The primary limitation of thoracic radiotherapy is radiation-induced lung injury. The administration of high-dose radiation to healthy lung tissue can induce alveolar damage, resulting in acute radiation pneumonitis (RP), a common radiotherapy-related adverse event with reported incidence rates ranging from 5% to 58% (3–5). Concurrently, immune-related pneumonia, such as checkpoint inhibitor pneumonitis (CIP), is relatively prevalent in lung cancer patients undergoing immunotherapy, with documented incidence rates ranging between 3% and 19% (6, 7). When radiotherapy is combined with immunotherapy, the synergistic effects of these modalities not only bolster antitumor efficacy but also exacerbate lung injury (8). Both RP and CIP can significantly impact patient quality of life and, in severe cases, can impede treatment progress or lead to fatal outcomes.

Chen et al. (9) investigated the impact of radiotherapy on pulmonary toxicity following immunotherapy and confirmed that lung V20 played a pivotal role as a risk factor for the development of SP, with statistical significance (95% CI: 1.41-8.66, P value = 0.007). This parameter is crucial for predicting pulmonary toxicity after combined treatment in lung cancer patients. Furthermore, various studies have highlighted that underlying lung conditions, smoking history, and other factors serve as predictive indicators of pulmonary toxicity in patients undergoing thoracic radiotherapy combined with immunotherapy (10, 11). Through advanced computational methods, radiomics extracts a number of quantitative features from medical images that can be analyzed with machine learning algorithms. This process significantly aids clinical decision-making by providing detailed, actionable insights (12, 13). Krafft et al. (14) utilized pretreatment CT images, extracted radiomic features from the ROI and integrated clinical and dosimetric parameters to construct a model for RP, achieving an AUC of 0.68. Additionally, Colen et al. (15) successfully constructed a model for predicting immune-related pneumonia with high accuracy (AUC: 1) by extracting radiomic features from thoracic CT images. However, these studies primarily focused on predicting pulmonary toxicity induced only by radiotherapy or immunotherapy. Nonetheless, the potential pulmonary toxicity resulting from radio-immunotherapy must not be underestimated. Regrettably, there is a dearth of research dedicated to utilizing radiomics to predict pulmonary toxicity associated with radiotherapy combined with immunotherapy. Consequently, developing a model capable of accurately predicting radiotherapy- and immunotherapy-related pneumonitis in lung cancer patients is a pressing need.

The objective of this research was to create a hybrid model combining radiomic features, deep image characteristics, and clinical information to forecast SP in patients with lung cancer who received immunotherapy and thoracic radiotherapy.

## 2 Materials and methods

In this research project, we focused on creating a comprehensive multimodal fusion model that combines various types of data - including deep image features, radiomics features, and clinical information. The goal was to use this model to accurately predict the occurrence of SP in patients with lung cancer who are undergoing thoracic radiotherapy in conjunction with immunotherapy. Our project involved several key steps: clinical information extraction module, feature extraction module including radiomic features and deep image features, and fusion of multimodal data prediction module.

### 2.1 Clinical information extraction

Retrospective data from 261 lung cancer patients who underwent thoracic radiotherapy combined with immunotherapy at the Harbin Medical University Cancer Hospital between January 2018 and May 2023 were collected. These data encompassed the clinical, immunological, and dosimetric parameters of the patients. Patients were selected based on specific criteria. The criteria for inclusion include: (1) aged ≥18 years; (2) had primary lung cancer confirmed by pathology; (3) received lung lesion radiotherapy combined with immunotherapy; (4) had complete dose-volume parameters and other information available; and (5) had a follow-up period exceeding 3 months postradiotherapy to acquire comprehensive pre- and posttreatment imaging data. The criteria for exclusion include: (1) active pulmonary infection; (2) pneumonia or abscess irrelevant to the tumor; (3) atelectasis or pleural effusion; and (4) loss to follow-up or incomplete imaging data. The study received approval from the hospital’s ethical review committee. Due to this research was a retrospective study, patient informed consent was not required.

### 2.2 Feature extraction

#### 2.2.1 CT scan and treatment implementation

A CT simulation positioning system is used to scan and position patients for radiotherapy treatment planning. The system has a scanning layer thickness of 5 mm to ensure accurate imaging. After the scanning process, the images are transferred to the pinnacle radiotherapy planning system. In this system, experienced radiotherapists carefully delineate the target area. GTV includes the main lung tumor and detectable metastatic lymph nodes on CT scans. Following GTV delineation, a 6 mm external expansion is applied for squamous cell carcinoma, while 8 mm is used for adenocarcinoma and small cell carcinoma to create the CTV. A 5 mm three-dimensional expansion is carried out from the CTV to create the PTV. A total irradiation dose of 50-70Gy/25-35f was administered to the PTV, with 95% of the dose covering 95% of the prescription volume. The maximum dose point was limited to 110% of the prescribed dose. During this process, various organs at risk, including the lungs, spinal cord, heart, and esophagus, are meticulously outlined. To ensure optimal treatment delivery, a conventional split-dose plan is utilized with the prescribed dose covering 95% of the PTV. Dosimetric constraints include bilateral lung V5 should be kept below 65%, V20 below 30%, V30 below 20%, with a MLD under 15 Gy. Additionally, the spinal cord’s maximum dose should not exceed 45 Gy, the heart’s V30 should be below 40% and V40 below 30%, with a mean dose under 26 Gy, and the esophagus should have a maximum dose below 105% of the prescribed dose and a V50 below 50%. Once target and organ delineations are completed, experienced physicists develop the treatment plan on the planning system, which is then reviewed and approved by the clinician before implementation. Patients undergo immunotherapy before, after, or concurrently with radiotherapy with PD-(L)1 inhibitors such as sintilimab, camrelizumab, pembrolizumab, toripalimab, atezolizumab, tislelizumab, durvalumab, slulimumab, and sugalizumab. Most patients receive a median of 4-6 cycles of immunodrug therapy, 3 weeks apart by intravenous infusion.

#### 2.2.2 Radiomic features extraction

The region of interest (ROI) comprises bilateral normal lung tissue, excluding the GTV, hilus, atelectasis, and thickened pleura. The DICOM-formatted image is imported into 3D Slicer, where an automatic segmentation algorithm is employed to identify the parenchyma of both lungs. Subsequently, manual adjustments are made to exclude nonlung tissues, resulting in a three-dimensional image of both lungs as the ROI (16, 17). A radiotherapist initially conducts the image segmentation process, which is then reviewed by a senior radiotherapist.

The image is standardized before extracting features. Feature extraction in radiomics is conducted using the PyRadiomics toolkit, which is an open-source tool compatible with the Python programming language. This toolkit facilitates the extraction of radiomic features from ROIs (18). Through this process, a comprehensive set of 107 radiomic features is obtained. These features include 14 shape-based histograms, which provide insights into the geometric properties of the ROIs, 18 first-order statistics (FOS) that describe basic intensity information, and 75 texture features that assess various statistical textures. In deep image feature extraction, training a deep learning (DL) model is computationally intensive and demands a substantial image dataset. Transfer learning facilitates DL models in transferring knowledge effectively. In this study, the original image data are converted to RGB format, cropped based on the label, and adjusted to a uniform size of 224x224. Data augmentation, such as horizontal image flipping, is employed to enhance the model’s generalizability and robustness. The training set’s diversity is increased by mirroring images horizontally, allowing the model to learn features from different perspectives and angles, enhancing its adaptability to varying input images.

#### 2.2.3 Deep image features extraction

The image data are normalized before deep image feature extraction to meet the input requirements of the model. The normalized data are fed into ResNet34 to extract dimensional image features. ResNet, a variant of convolutional neural network (CNN), excels in local feature representation and places greater emphasis on image details, making it more effective than deep neural networks (DNNs) in these aspects:


(1) y=F(x,W)+x

where x is the input, y is the output, and ℱ(x,W) is the residual function, which represents a nonlinear mapping involving multiple layers (each layer using weights W ). For medical images depicting lesions, ResNet can better highlight subtle differences in malignancy. The structural parameters of ResNet34 is shown in **Table 1**.

**Table 1:** The structural parameters of ResNet34.

Index	Layer Name	Parameters
1	Convolutional Layer	7 × 7, 64, stride=2
2	Pooling Layer	3 × 3 max pool, stride=2
3	Convolutional Layers	3 $\times \{\begin{array}{c} 3\times 3,\;64\\ 3\times 3,\;64 \end{array}$
4	Convolutional Layers	4 $\times \{\begin{array}{c} 3\times 3,\;128\\ 3\times 3,\;128 \end{array}$
5	Convolutional Layers	6 $\times \{\begin{array}{c} 3\times 3,\;256\\ 3\times 3,\;256 \end{array}$
6	Convolutional Layers	3 $\times \{\begin{array}{c} 3\times 3,\;512\\ 3\times 3,\;512 \end{array}$
7	Pooling Layer	Average pool, 512-D output

### 2.3 Fusion of multimodal data prediction

**Figure 1:**
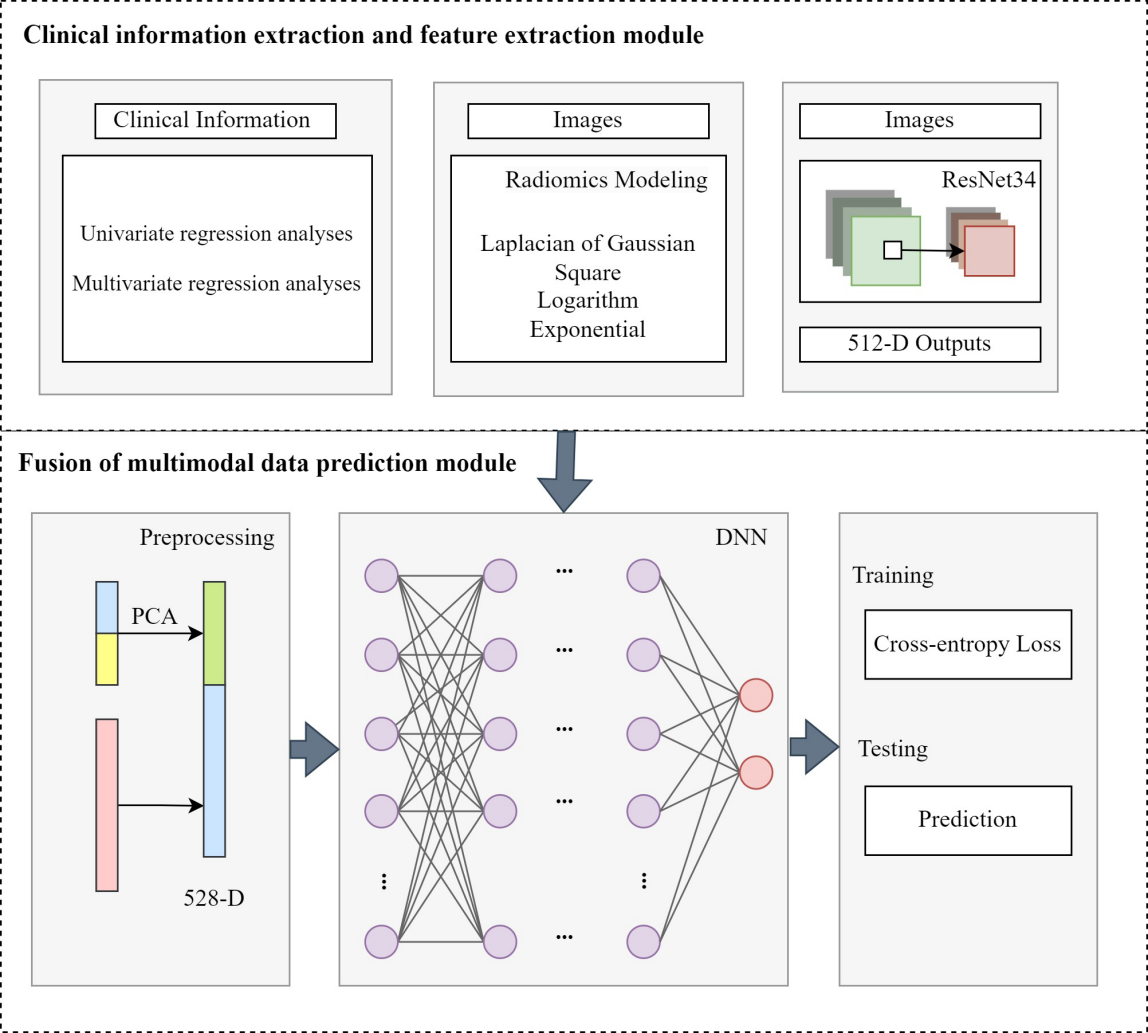
The structure of fusion of multimodal data prediction module.


Univariate and multivariate regression analyses of clinical baseline characteristics are performed to identify 12 clinical indicators potentially associated with SP. After extracting multimodal features, we integrate them and feed them into a DNN-based prediction module, as illustrated in the **Figure 1**. The clinical data, traditional radiomic features, and deep image features are normalized and dimensionally reduced using principal component analysis (PCA) to enhance computational efficiency and reduce storage requirements. The dimensionally reduced data are concatenated and fed into a fully connected layer for final prediction. The formula is as followed:

(2) $$F\;=\;concat\left(F_{c},\;F_{t},\;F_{d}\right)$$

(3) $$z_{1}\;=\;\sigma_{1}\left(W_{1}\left(F\right)+b_{1}\right)\;\;$$

(4) $$z_{2}\;=\;\sigma_{2}\left(W_{2}\left(z_{1}\right)+b_{2}\right)\;$$

(5) $$z_{L}\;=\;\sigma_{L}\left(W_{L}\left(z_{L-1}\right)+b_{L}\right)\;$$

where ${\text{F}}_{\text{c}}$ is the clinical features, ${\text{F}}_{\text{t}}$ is the radiomic features and ${\text{F}}_{\text{d}}$ is the deep image features. L is the number of fully connected layers. ${\text{W}}_{\text{i}}$ represents the weight matrix, ${\text{b}}_{\text{i}}$ is the bias vector and ${\text{σ}}_{\text{i}}$ is the activation function for i-th layer. In the study, we used LeakyReLU function as the σ . The loss function we used as follows:


(6) $$Loss\;=\;-\sum^{}y_{i}log{\hat{y}}_{i}+\;\left(1-y_{i}\right)log(1-{\hat{y}}_{i})$$

where ${\hat{y}}_{i}$ is the label, ${\text{y}}_{\text{i}}$ is the prediction of our method.

## 3 Results

### 3.1 Datasets

This study included 261 lung cancer patients received a combination of thoracic radiotherapy and immunotherapy at Harbin Medical University Cancer Hospital between January 2018 and May 2023. Among them, 36.02% (94 patients) experienced SP, with 6.13% (16 patients) classified as G3+ pneumonitis. **Table 2** presents the baseline characteristics of the patients. In the SP group, the median age was 61 years, with males comprising 81.9% of the group. Additionally, 88.3% of the patients had a PS score ranging from 0 to 1, and 53.2% had a history of smoking. Squamous cell carcinoma was the most prevalent histological type at 51.1%, while SCLC accounted for 25.5%. In terms of disease staging, T2-stage disease was present in 36.2% of patients, while N2-stage disease was noted in 63.8% of individuals. Importantly, 71.3% of the patients received a radiotherapy dose of 60 Gy or higher, and 78.7% underwent 4 or more cycles of chemotherapy. At the time of patient follow-up, the median number of immune drug cycles administered was 4. The most commonly used immunotherapy drug was camrelizumab (23.4%), followed by tislelizumab (20.2%). Notably, 12.8% of patients received combination immunotherapy drugs. Twelve clinical indicators potentially linked to SP were identified, including the ECOG PS score, T stage, N stage, radiotherapy dose, number of immunotherapy cycles, preradiotherapy NLR, preradiotherapy PLR, preradiotherapy LMR, preradiotherapy SII, V5, V20, and MLD. Based on the univariate analysis, the PS score (HR: 3.556, 95% CI: 1.270-9.955, P=0.016), T-stage (HR: 1.558, 95% CI: 1.198-2.026, P=0.001), and PLR before radiotherapy (HR: 1.003, 95% CI: 1.000-1.006, P=0.028) were significantly linked to the occurrence of SP. The multivariate analysis indicated that PS and T-stage were independent predictors for SP (HR: 3.322, 95% CI: 1.148-9.615, P=0.027; HR: 1.501, 95% CI: 1.147-1.965, P=0.003). Refer to **Table 3** for the results of both univariate and multivariate analyses on SP.

**Table 2:** Baseline characteristics.

Characteristics	Non-symptomatic pneumonia(n=167)	Symptomatic pneumonia(n=94)	All (n=261)	P value
Age, (y)				0.698
<61	77 (46.1)	41 (43.6)	118 (45.2)	
≥61	90 (53.9)	53 (56.4)	143 (54.8)	
Gender				0.503
Male	131 (78.4)	77 (81.9)	208 (79.7)	
Female	36 (21.6)	17 (18.1)	53 (20.3)	
ECOG PS				0.011
0-1	161 (96.4)	83 (88.3)	244 (93.5)	
≥2	6 (3.6)	11 (11.7)	17 (6.5)	
Smoker History				0.793
No	81 (48.5)	44 (46.8)	125 (47.9)	
Yes	86 (51.5)	50 (53.2)	136 (52.1)	
Smoking Index				0.273
<400	97 (58.1)	48 (51.1)	145 (55.6)	
≥400	70 (41.9)	46 (48.9)	116 (44.4)	
Tumor Histology				0.269
Squamous Cell Carcinoma	76 (45.5)	48 (51.1)	124 (47.5)	
Adenocarcinoma	34 (20.4)	18 (19.1)	52 (19.9)	
Small Cell Lung Cancer	55 (32.9)	24 (25.5)	79 (30.3)	
Other	2 (1.2)	4 (4.3)	6 (2.3)	
T Stage				0.000
1	63 (37.7)	16 (17.0)	79 (30.3)	
2	54 (32.3)	34 (36.2)	88 (33.7)	
3	34 (20.4)	30 (31.9)	64 (24.5)	
4	16 (9.6)	14 (14.9)	30 (11.5)	
N Stage				0.779
0	32 (19.2)	11 (11.7)	43 (16.5)	
1	4 (2.4)	4 (4.3)	8 (3.1)	
2	91 (54.5)	60 (63.8)	151 (57.9)	
3	40 (24.0)	19 (20.2)	59 (22.6)	
M Stage				0.937
0	104 (62.3)	59 (62.8)	163 (62.5)	
1	63 (37.7)	35 (37.2)	98 (37.5)	
Radiotherapy Dose				0.236
<60Gy	37 (22.2)	27 (28.7)	64 (24.5)	
≥60Gy	130 (77.8)	67 (71.3)	197 (75.5)	
Radiotherapy Fractions				0.356
<30f	36 (21.6)	25 (26.6)	61 (23.4)	
≥30f	131 (78.4)	69 (73.4)	200 (76.6)	
Chemotherapy Cycles				0.596
<4	31 (18.6)	20 (21.3)	51 (19.5)	
≥4	136 (81.4)	74 (78.7)	210 (80.5)	
Cycles of Immunotherapy	4.00 (2.00-6.00)	4.00 (2.00-5.00)	4.00 (2.00-6.00)	0.474
Type of immunotherapy				0.158
Sintilimab	19 (11.4)	7 (7.4)	26 (10.0)	
Camrelizumab	52 (31.1)	22 (23.4)	74 (28.4)	
Pembrolizumab	11 (6.6)	11 (11.7)	22 (8.4)	
Toripalimab	7 (4.2)	10 (10.6)	17 (6.5)	
Atezolizumab	11 (6.6)	4 (4.3)	15 (5.7)	
Tislelizumab	23 (13.8)	19 (20.2)	42 (16.1)	
Durvalumab	19 (11.4)	6 (6.4)	25 (9.6)	
Serplulimab	6 (3.6)	3 (3.2)	9 (3.4)	
Sugemalimab	2 (1.2)	0 (0.0)	2 (0.8)	
Combination immunotherapy drugs	17 (10.2)	12 (12.8)	29 (11.1)	
NLR1	2.40 (1.70-3.50)	2.70 (1.80-4.35)	2.50 (1.70-3.75)	0.070
NLR2	4.30 (2.90-7.50)	4.55 (2.98-7.60)	4.50 (2.95-7.55)	0.607
NLR3	4.20 (2.60-6.60)	4.70 (3.10-7.35)	4.40 (2.90-6.95)	0.214
PLR1	122.10 (97.90-175.60)	146.10 (98.98-212.68)	126.70 (97.90-186.45)	0.078
PLR2	219.30 (163.00-310.90)	224.85 (159.65-327.30)	219.30 (160.65-315.50)	0.795
PLR3	239.80 (157.30-357.80)	253.60 (147.60-416.45)	241.80 (156.25-371.00)	0.667
LMR1	3.70 (2.60-5.00)	3.05 (2.20-4.30)	3.40 (2.40-4.60)	0.016
LMR2	1.70 (1.20-2.30)	1.70 (1.10-2.23)	1.70 (1.10-2.30)	0.550
LMR3	1.90 (1.20-2.70)	1.60 (1.20-2.60)	1.80 (1.20-2.60)	0.504
SII1 (x 10^9^/L)	486.40 (331.30-850.70)	608.65 (372.10-1013.95)	519.70 (350.15-912.90)	0.090
SII2 (x 10^9^/L)	806.20 (531.70-1349.40)	870.10 (540.60-1459.45)	826.10 (539.75-1371.95)	0.949
SII3 (x 10^9^/L)	898.20 (524.00-1428.60)	944.85 (537.85-1745.35)	940.50 (533.65-1550.55)	0.417
V_5_	40.94 (33.37-46.00)	41.48 (33.99-49.04)	41.00 (33.69-46.75)	0.281
V_20_	22.17 (18.02-25.00)	22.05 (18.69-26.00)	22.10 (18.11-25.06)	0.257
V_30_	16.35 (12.78-19.21)	16.43 (12.98-20.05)	16.38 (12.85-19.70)	0.312
MLD (Gy)	12.07 (10.06-13.88)	12.15 (10.21-14.93)	12.13 (10.11-14.11)	0.359

Combination immunotherapy drugs: Using more than two immunological drugs in combination; NLR1, NLR before radiotherapy; NLR2, NLR in radiotherapy; NLR3, NLR after radiotherapy; PLR1, PLR before radiotherapy; PLR2, PLR in radiotherapy; PLR3, PLR after radiotherapy; LMR1, LMR before radiotherapy; LMR2, LMR in radiotherapy; LMR3, LMR after radiotherapy; SII1, SII before radiotherapy; SII2, SII in radiotherapy; SII3, SII after radiotherapy; V5, Lung volume at least 5 Gy irradiated; V20, Lung volume at least 20 Gy irradiated; V30, Lung volume at least 30 Gy irradiated.

**Table 3:** Results of both univariate and multivariate analyses on SP.

	Univariate analysis		Multivariate analysis	
	HR (95% CI)	P value	HR (95% CI)	P value
Age	1.022 (0.990-1.055)	0.185		
Gender	0.803 (0.423-1.526)	0.504		
ECOG PS	3.556 (1.270-9.955)	**0.016**	3.322 (1.148-9.615)	**0.027**
Smoker History	0.934 (0.563-1.550)	0.793		
Smoking Index	0.753 (0.453-1.251)	0.274		
Tumor Pathology	0.923 (0.701-1.215)	0.566		
T-Stage	1.558 (1.198-2.026)	**0.001**	1.501 (1.147-1.965)	**0.003**
N-Stage	1.111 (0.847-1.457)	0.446		
M-Stage	0.979 (0.581-1.651)	0.937		
Radiotherapy Dose	1.416 (0.795-2.521)	0.238		
Radiotherapy Fractions	1.318 (0.733-2.373)	0.357		
Chemotherapy Cycles	1.186 (0.632-2.225)	0.596		
Cycles of Immunotherapy	0.971 (0.909-1.037)	0.374		
Type of immunotherapy	1.032 (0.945-1.127)	0.485		
NLR1	1.071 (0.987-1.162)	0.100		
NLR2	1.010 (0.952-1.072)	0.741		
NLR3	1.018 (0.988-1.049)	0.238		
PLR1	1.003 (1.000-1.006)	**0.028**	1.003 (1.000-1.006)	0.059
PLR2	1.000 (0.998-1.002)	0.924		
PLR3	1.000 (0.999-1.002)	0.694		
LMR1	0.920 (0.837-1.012)	0.086		
LMR2	1.015 (0.834-1.234)	0.884		
LMR3	0.988 (0.868-1.125)	0.856		
SII1 (x 10^9^/L)	1.000 (1.000-1.001)	0.124		
SII2 (x 10^9^/L)	1.000 (1.000-1.000)	0.653		
SII3 (x 10^9^/L)	1.000 (1.000-1.000)	0.170		
V_5_	1.019 (0.994-1.045)	0.128		
V_20_	1.038 (0.992-1.087)	0.106		
V_30_	1.040 (0.989-1.094)	0.123		
MLD (Gy)	1.059 (0.982-1.143)	0.139		

HR, Hazard ratio; CI, Confidence interval; NLR1, NLR before radiotherapy; NLR2, NLR in radiotherapy; NLR3, NLR after radiotherapy; PLR1, PLR before radiotherapy; PLR2, PLR in radiotherapy; PLR3, PLR after radiotherapy; LMR1, LMR before radiotherapy; LMR2, LMR in radiotherapy; LMR3, LMR after radiotherapy; SII1, SII before radiotherapy; SII2, SII in radiotherapy; SII3, SII after radiotherapy; V5, Lung volume at least 5 Gy irradiated; V20, Lung volume at least 20 Gy irradiated; V30, Lung volume at least 30 Gy irradiated. Bold values mean P < 0.05 indicates statistical significance.

### 3.2 Evaluation metrics

#### 3.2.1 SP outcome evaluation

Thoracic CT scans are recommended for regular monitoring in conjunction with clinical indicators. The first thoracic CT scan is scheduled when the radiotherapy dose reaches 40 Gy, followed by another scan one month after completing radiotherapy. Monitoring continued for a minimum of three months or until G2+ SP is detected. Pulmonary toxicity diagnosis relies on the patient’s clinical symptoms and imaging findings, which are confirmed collaboratively by a senior radiotherapist and an imaging specialist. Concerning changes seen on imaging may consist of ground-glass opacities, as well as flaky or flocculent mixed nodules within the radiotherapy area. Symptoms observed in clinical settings encompass dry cough, fever, and difficulty breathing. Pulmonary toxicity is assessed following CTCAE version 5.0 guidelines, with SP diagnosed at Grade 2 or above.

#### 3.2.2 Model evaluation

Data analysis was conducted utilizing SPSS 26.0 software. For analyzing categorical data, either the χ^2^ test or Fisher’s exact test was applied. For measurement data, the independent sample t-test or Mann–Whitney U test was utilized. To identify independent predictors of SP occurrence, both univariate and multivariate regression analyses were performed. Additionally, ROC curves were created, and several metrics, including the AUC, sensitivity, specificity, accuracy, positive predictive value, F1 score, MCC, and kappa value, were calculated to evaluate and compare the predictive capabilities of the models. Differences between the models were determined using the DeLong test, with statistical significance set at P < 0.05.

### 3.3 Comparison results

To validate the predictive capability of our proposed algorithm for symptomatic pneumonitis caused by lung cancer, we compared it with the classical machine learning algorithm RF and the classical deep learning algorithm. Additionally, we discussed the impact of multimodal data on the algorithm.

#### 3.3.1 Radiomics model predictive performance

**Figure 2:**
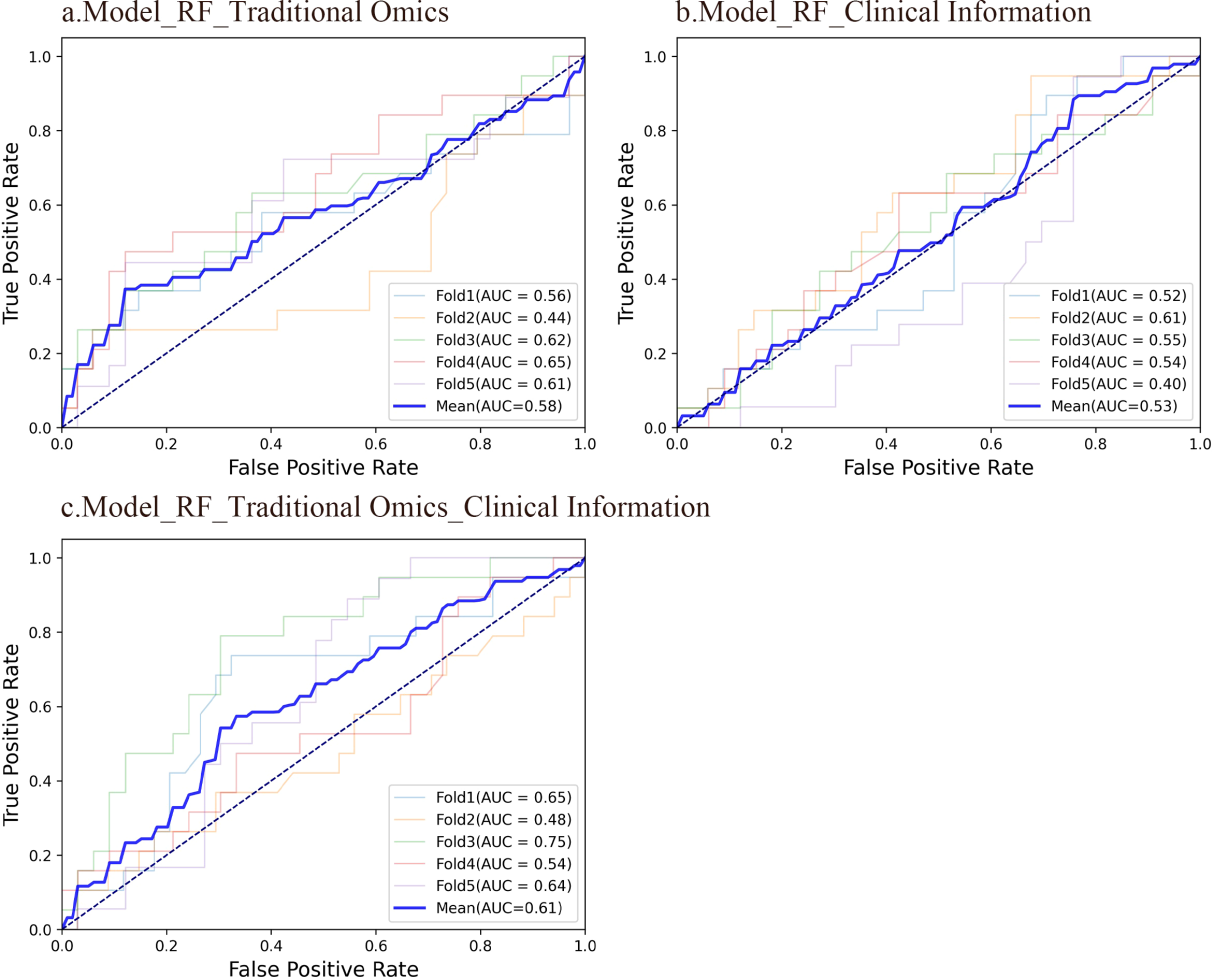
ROC curves of the three RF models. **(A)** An RF model using radiomic features had an average AUC of 0.58. 95%CI: 0.523-0.628. **(B)** An RF model using clinical information had an average AUC of 0.53. 95%CI: 0.479-0.572. **(C)** An RF model integrating conventional radiomic features with clinical data had an average AUC of 0.61. 95%CI: 0.566-0.652.

RF is a model composed of multiple independently trained decision trees. These trees are constructed by randomly selecting samples and features, with predictions made through voting or averaging. The study found that the AUC of the RF model using radiomic features was 0.576, indicating moderate predictive performance. In comparison, the RF model using only clinical information had a lower AUC of 0.525. However, when integrating radiomics characteristics with clinical data, the AUC of the RF model showed a notable increase to 0.611, demonstrating better predictive accuracy. Despite the enhanced performance of the RF model with combined features, it was noted that the overall predictive capability was still not optimal. The ROC curves of the three RF models are illustrated in **Figure 2**.

**Figure 3:**
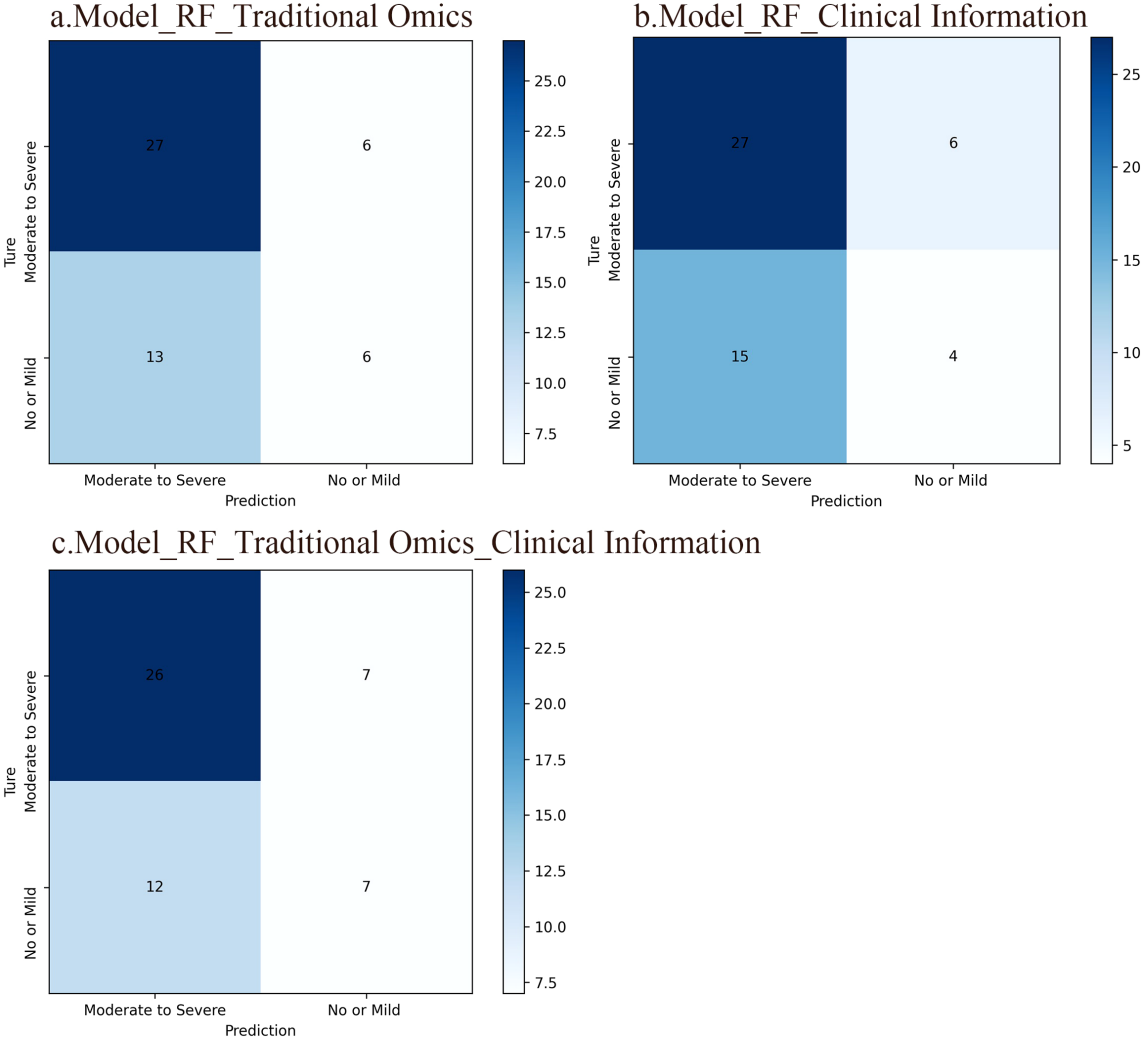
Confusion matrices of the three RF models. The rows in the matrix represent the model’s predicted outcomes, while the columns represent actual SP occurrences. Each column/row of the matrix represents the number of patients in the actual/predicted category. **(A)** An RF model based on traditional radiomic features. The accuracy of this model was 63.5%. **(B)** An RF model based on clinical information and the accuracy of this model was 59.6%. **(C)** An RF model integrating conventional radiomic features with clinical data, which accuracy was 63.5%.

The confusion matrix serves as a crucial tool in machine learning to evaluate classification model effectiveness. It illustrates the correlation between the model’s forecasts in various groups and the true labels in matrix structure. Findings reveal that the RF model developed with radiomic features obtained an accuracy rate of 63.5%. The RF model established with clinical data achieved an accuracy of 59.6%. In addition, the RF model generated by merging radiomic features and clinical information reached an accuracy rate of 63.5%. In contrast to the radiomics feature model, the model relying solely on clinical data displayed a slightly lower accuracy. Interestingly, the integration of radiomic characteristics with clinical data in the model yielded the same precision as the model utilizing only radiomic features. This emphasizes the potential performance enhancement gained by combining distinct feature types, potentially due to the complementary information provided by these two feature sets. The confusion matrices of the three RF models are illustrated in **Figure 3**.

**Figure 4:**
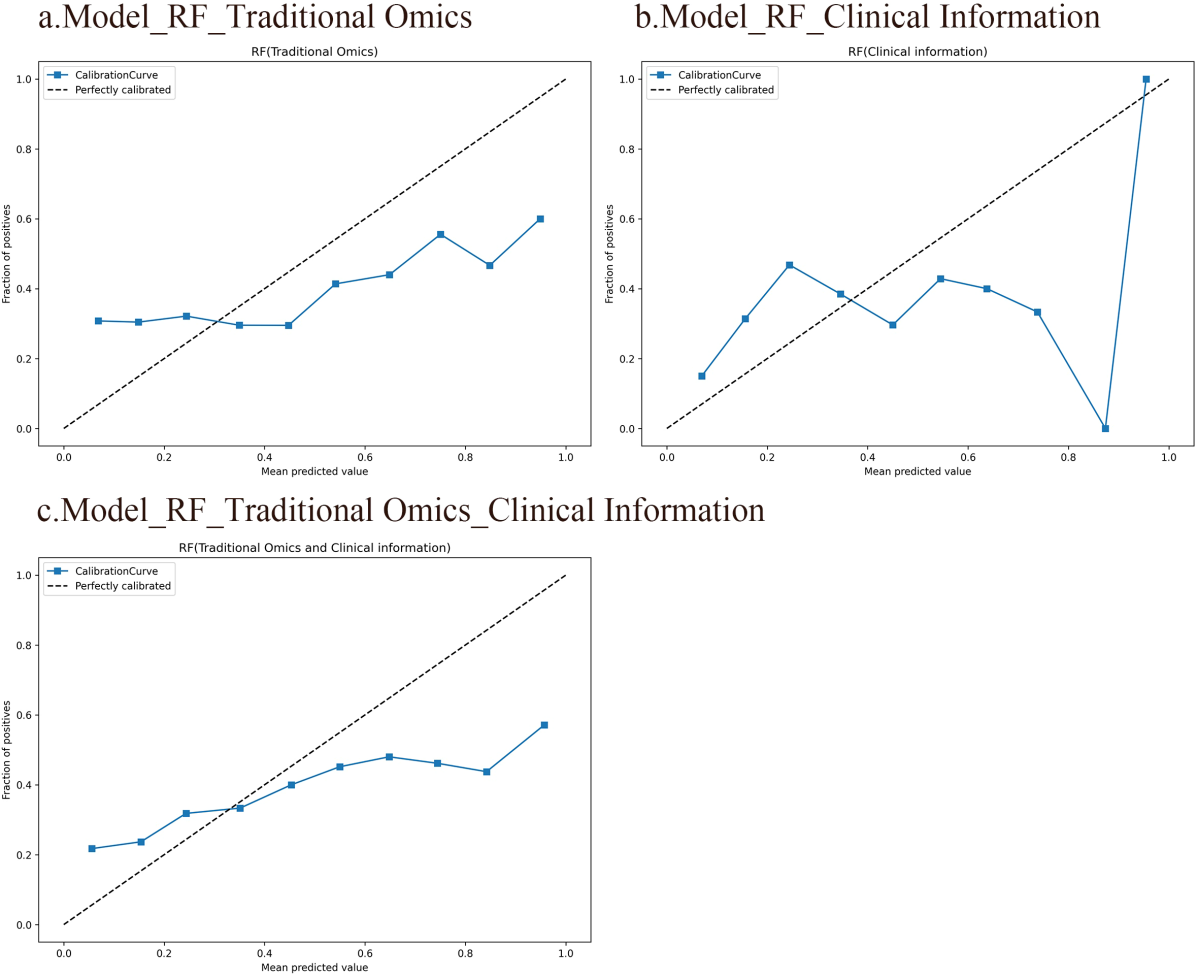
Calibration curves of the three RF models. The horizontal axis represents the predicted probabilities of the model, while the vertical axis represents the accuracy. Ideally, the calibration curve should closely align with the 45° diagonal line, indicating perfect consistency between the model’s predicted and actual probabilities of events occurring. **(A)** An RF model based on traditional radiomic features. **(B)** An RF model based on clinical information. **(C)** An RF model integrating conventional radiomic features with clinical data.

A calibration curve shows the link between the observed and predicted probabilities. Ideally, the calibration curve should closely align with the 45° diagonal line, indicating perfect consistency between the model’s predicted and actual probabilities of events occurring. The results indicate that the RF model, which integrates radiomic features with clinical information, is highly consistent between the predicted and observed probabilities for SP and non-SP. In contrast, the calibration curves of the other two models deviate significantly from the diagonal line, suggesting less accurate predictions. **Figure 4** displays the calibration curves for all three RF models.

#### 3.3.2 Predictive performance of the DL model

Our approach utilizes multimodal data combined with ResNet34 for prediction. In addition to RF, we also compared with DNN. DNNs are specialized neural network structures designed for processing images and visual data, typically comprising multiple convolutional and pooling layers to create a deep architecture. The pretrained ResNet34 encoder is utilized for extracting deep image features, which are high-level abstract features obtained from image data through DL techniques that capture information such as shape and object categories.

**Figure 5:**
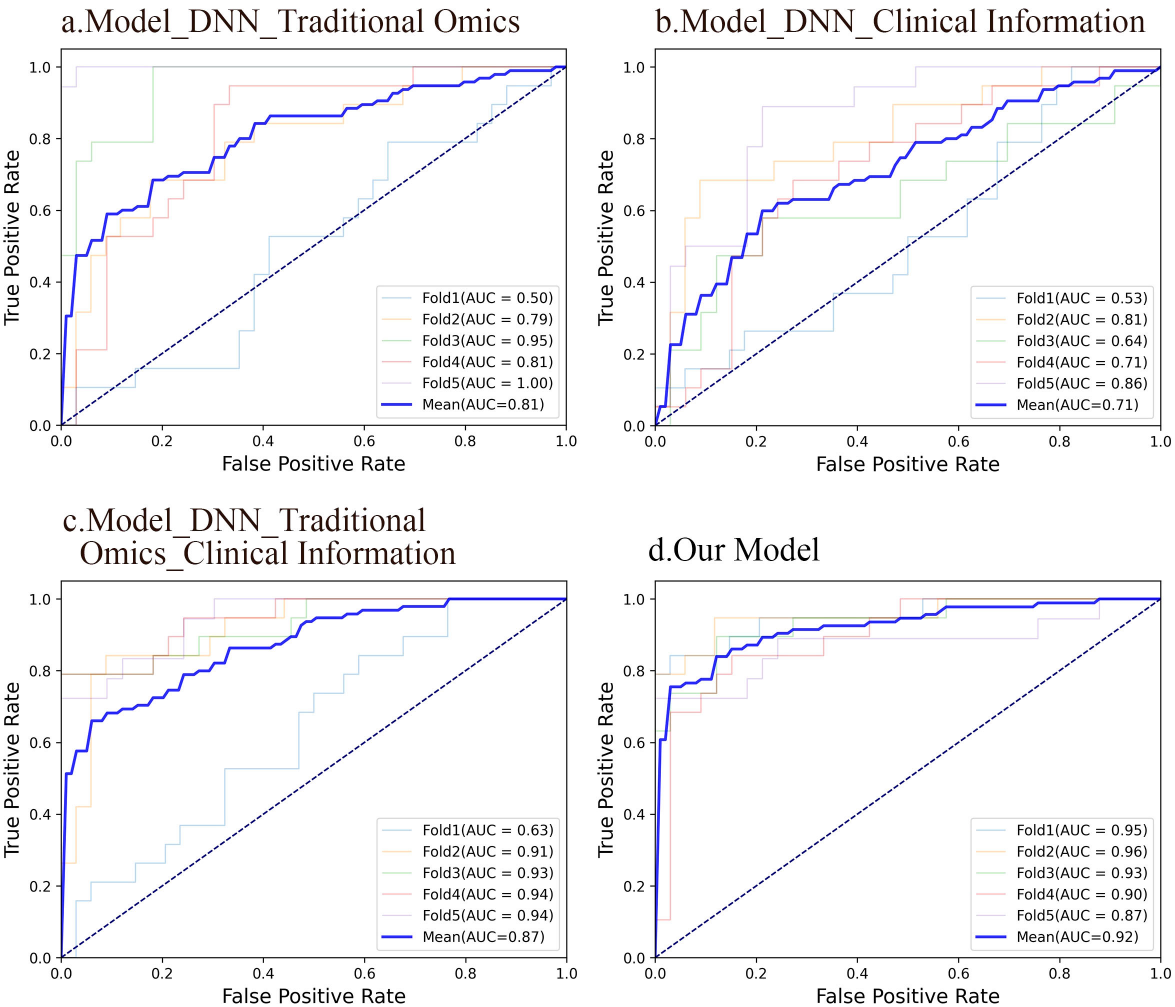
ROC curves of the four DNN models. **(A)** A DNN model using traditional radiomic features had an average AUC of 0.81. 95%CI: 0.786-0.832. Significance level: <0.001. **(B)** A DNN model using clinical information had an average AUC of 0.71. 95%CI: 0.682-0.753. Significance level: <0.001. **(C)** A DNN model integrating conventional radiomic features with clinical data had an average AUC of 0.87. 95%CI: 0.845-0.896. Significance level: <0.001. **(D)** A fusion model based on deep image features, radiomic features, and clinical information had an average AUC of 0.92. 95%CI: 0.902-0.945. Significance level: <0.001.

In this study, dimensionally reduced radiomic features, clinical data, and depth image features were entered into the fully connected layer to construct deep neural network models based on radiomic features (AUC 0.811), clinical information (AUC 0.711), and combined radiomic with clinical characteristics (AUC 0.872). Furthermore, integrating deep image features with radiomic and clinical characteristics resulted in a multidimensional fusion model with an AUC of 0.922. Notably, the multidimensional fusion model superior the other three models significantly. Similarly, the model combining radiomics features with clinical information exhibited greater accuracy than did the individual models, with the radiomics-based model outperforming the clinical information-based model. **Figure 5** shows the ROC curves of the four DNN models.

**Figure 6:**
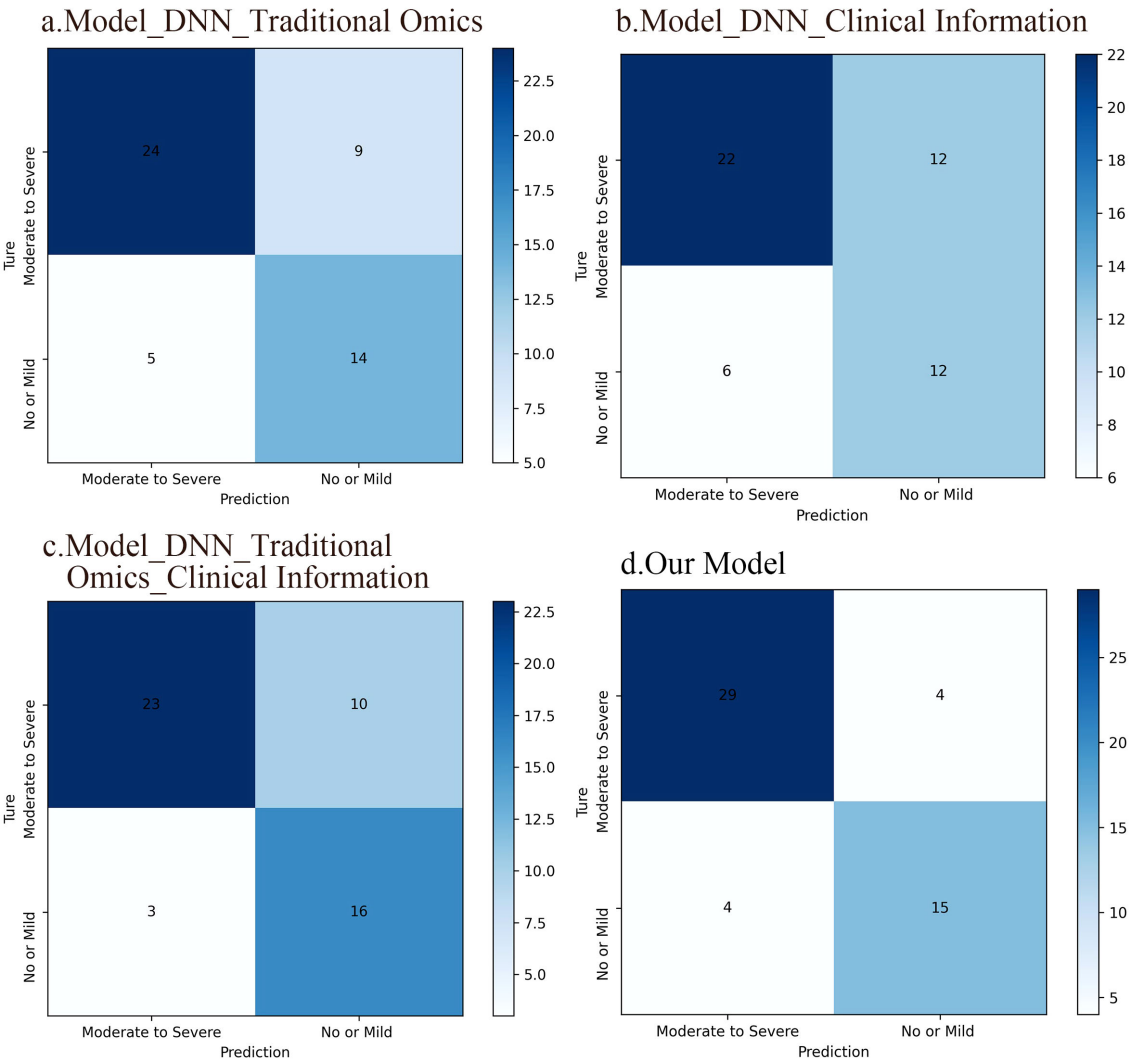
Confusion matrices of the four DNN models. **(A)** A DNN model based on traditional radiomic features, which accuracy was 73.1%. **(B)** A DNN model based on clinical information and the accuracy of this model was 65.4%. **(C)** A DNN model integrating conventional radiomic features with clinical data. The accuracy of this model was 75%. **(D)** A fusion model based on deep image features, radiomic features, and clinical information, which accuracy was 84.6%.

The confusion matrix revealed that the DNN model leveraging radiomic features achieved an accuracy of 73.1%, while the DNN model relying on clinical information attained 65.4% accuracy. Combining radiomics features with clinical data increased the accuracy to 75.0%. In comparison, the fusion model integrating deep image features, radiomics features, and clinical information achieved an accuracy of 84.6%. Clearly, our multidimensional fusion model demonstrates the highest accuracy, demonstrating the benefits of a comprehensive approach incorporating depth image features, radiomics features, and clinical information. Although models based solely on radiomic features perform well in classification tasks, opportunities for improvement remain. Significantly, integrating conventional radiomic features with clinical data led to better accuracy than using each model separately. **Figure 6** illustrates the confusion matrices of the four DNN models.

**Figure 7:**
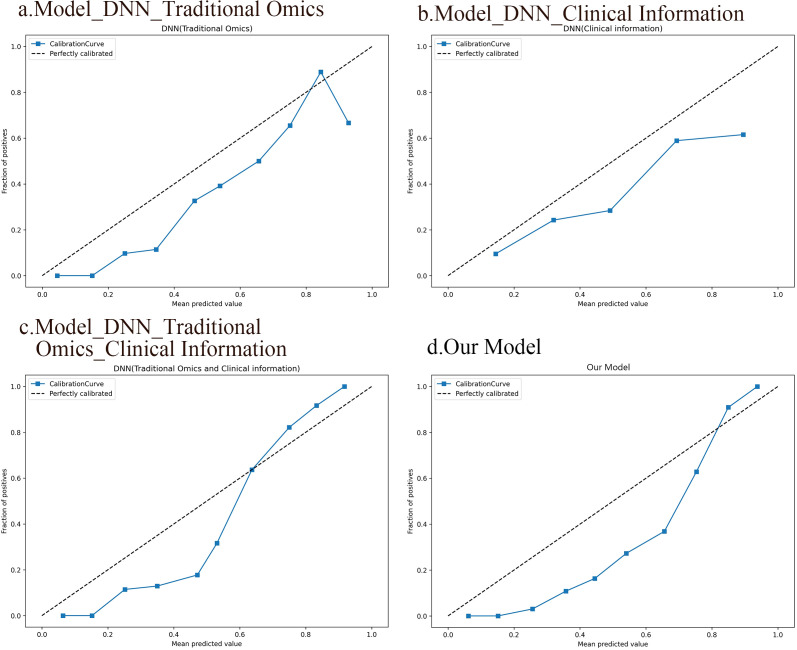
Calibration curves of the four DNN models. **(A)** A DNN model based on traditional radiomic features. **(B)** A DNN model based on clinical information. **(C)** A DNN model integrating conventional radiomic features with clinical data. **(D)** A fusion model based on deep image features, radiomic features, and clinical information.

The results from the calibration curve show a significant correlation between the anticipated probabilities of SP and non-SP when utilizing the integrated model that includes deep image characteristics, radiomics features, clinical data, and the observed probabilities. **Figure 7** displays the calibration curves for the four DNN models.

#### 3.3.3 Comparison of the prediction performances of different models

We conducted a comparison of seven different models and revealed that the fusion model incorporating deep image features, radiomics features, and clinical information significantly outperformed the other models (AUC 0.922, sensitivity 0.879, specificity 0.789). Combining f radiomic features with clinical information yielded better results than did the individual models (AUC 0.872, sensitivity 0.697, specificity 0.842) among the deep neural network models. In contrast, the RF classifier models, whether individual or combined, exhibited lower performance than did the DL models. Notably, both models utilizing radiomic features, DL and RF, demonstrated superior performance comparison with models relying solely on clinical data. This suggests that radiomic features have the capacity to capture intricate high-level characteristics that go beyond conventional clinical data. Additionally, the DeLong test confirmed that the DL models significantly outperformed the RF models (P<0.001), with the multidimensional fusion model incorporating deep image features, radiomics features, and clinical information showing a substantial enhancement in performance compared to the DL model (P<0.001). Detailed results of the seven models, including measures such as AUC, sensitivity, specificity, accuracy, positive predictive value, F1 score, MCC, and kappa value, can be found in **Table 4**.

**Table 4:** Performances evaluation sheet for all models.

	AUC (95%CI)	Sensitivity	Specificity	Accuracy	Precision	F1 score	MCC	Kappa	P value
RF_Traditional Omics	0.576 (0.523-0.628)	0.818	0.316	0.635	0.675	0.740	0.153	0.145	–
RF_Clinical Information	0.525 (0.479-0.572)	0.818	0.211	0.596	0.643	0.720	0.035	0.032	–
RF_Traditional Omics_Clinical Information	0.611 (0.566-0.652)	0.788	0.368	0.635	0.684	0.732	0.170	0.165	–
DNN_Traditional Omics	0.811 (0.786-0.832)	0.727	0.737	0.731	0.828	0.774	0.450	0.444	<0.001^a^
DNN_Clinical Information	0.711 (0.682-0.753)	0.647	0.667	0.654	0.786	0.710	0.299	0.291	<0.001^b^
DNN_Traditional Omics_Clinical Information	0.872 (0.845-0.896)	0.697	0.842	0.750	0.885	0.800	0.519	0.500	<0.001^c^
Our Model	0.922 (0.902-0.945)	0.879	0.789	0.846	0.879	0.879	0.668	0.668	<0.001^d^

a The AUC of a DNN model using traditional radiomic features versus the AUC of an RF model using traditional radiomic features by Delong test, P < 0.001.

b The AUC of a DNN model using clinical data versus the AUC of an RF model using clinical data by Delong test, P < 0.001.

c The AUC of a DNN model integrating conventional radiomic features with clinical data versus the AUC of an RF model integrating conventional radiomic features with clinical data by Delong test, P < 0.001.

d The AUC of our model versus the AUC of DNN models by Delong test, P < 0.001.

**Figure 8:**
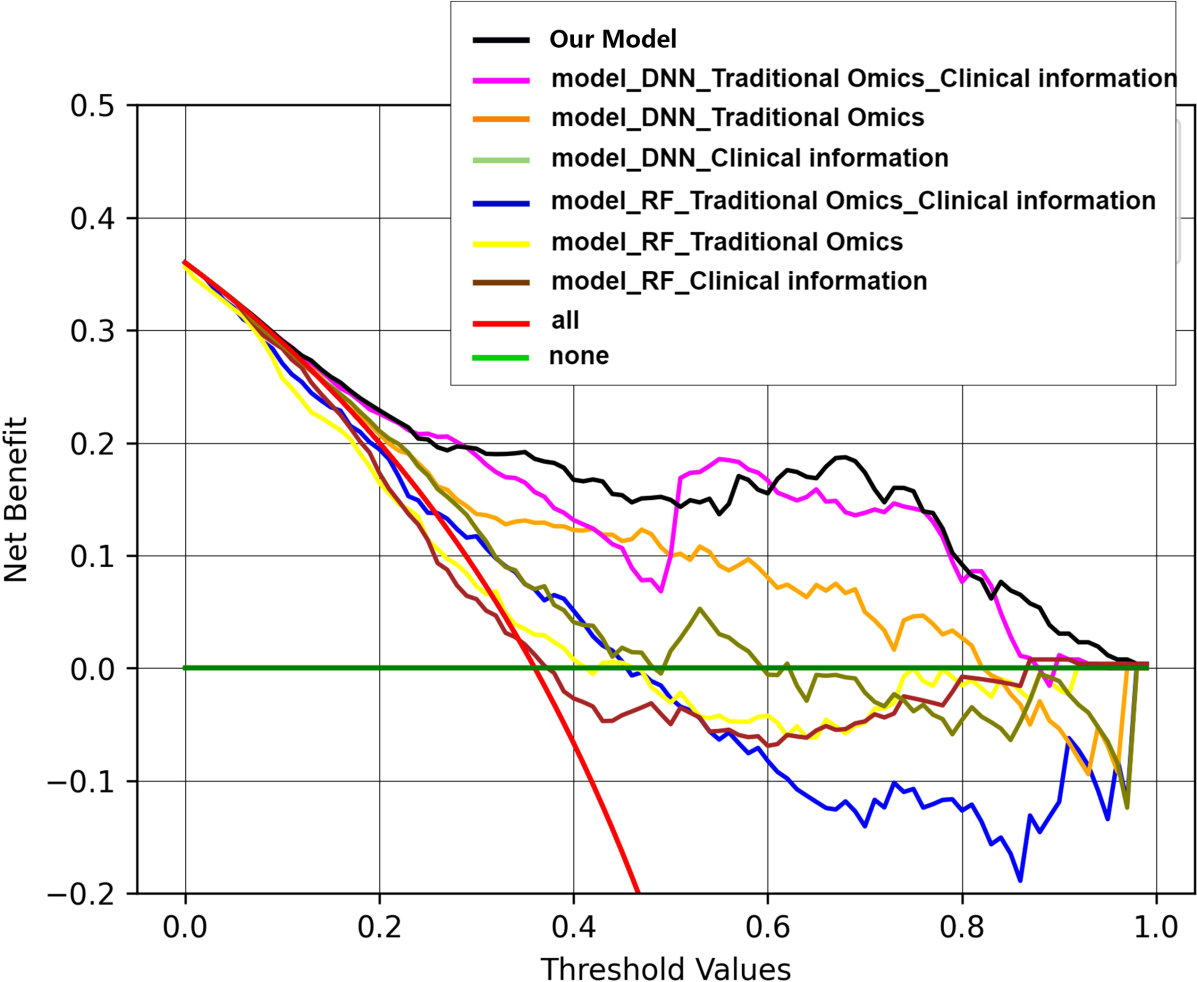
Decision curves of all the models. Within the DCA, the horizontal axis delineates the threshold range, while the vertical axis illustrates the net benefit. Results of the DCA show that the fusion model integrating deep image features, radiomics features, and clinical information outperforms other models in terms of net benefits across diverse decision thresholds.

Decision curve analysis (DCA) serves as a valuable tool for assessing predictive models, particularly in clinical decision-making contexts. Our utilization of DCA aimed to assess how different models impact clinical treatment decisions. Within the DCA, the horizontal axis delineates the threshold range, while the vertical axis illustrates the net benefit. Notably, results of the DCA show that the fusion model integrating deep image features, radiomics features, and clinical information outperforms other models in terms of net benefits across diverse decision thresholds. **Figure 8** shows the decision curves for all the models.

## 4 Discussion

In our research, we effectively created a predictive model for SP by integrating DL techniques with clinical information based on preradiotherapy CT imaging. This research represents a novel approach utilizing radiomics data to predict early-onset radiotherapy-immunotherapy-induced pneumonia in lung cancer patients undergoing combined radiotherapy and immunotherapy treatment.

### 4.1 SP occurrence rate analysis

The KEYNOTE-799 trial investigated the safety profile of pembrolizumab when used together with concurrent chemoradiotherapy for patients with inoperable LA-NSCLC. Findings showed that the occurrence of any-grade pneumonitis in both groups were 37.5% and 26.4%, while rates of G3+ pneumonitis were 8.0% and 6.9%, respectively (19). In real-world scenarios, patients often present complex conditions, such as undergoing multiple treatment cycles, having underlying illnesses, or being elderly. These individuals frequently harbor pneumonia-related risk factors, leading to their exclusion from clinical trials. In a retrospective study encompassing lung cancer patients receiving durvalumab immunoconsolidation postchemoradiotherapy across 10 institutions, 63.5% of patients developed pneumonitis during immunoconsolidation therapy, with SP accounting for 33.7% and grade 3 pneumonitis accounting for 8% (10). A real-world Japanese investigation of pneumonitis occurrence during durvalumab consolidation postchemoradiotherapy in NSCLC patients revealed an occurrence of any-grade pneumonitis of 83%, an incidence of SP of 34%, an incidence of G3+ pneumonitis of 7%, and a fatal pneumonitis rate of 1% (20). In this study, any-grade pneumonitis accounted for 76.63%, SP accounted for 36.02%, and grade 3 or above pneumonitis accounted for 6.13%, aligning closely with Japanese real-world research data but surpassing clinical trial outcomes. This finding indicates the critical importance of tailored prevention and management strategies for radiotherapy-immune-related pulmonary toxicity in diverse patient populations. A recent extensive meta-analysis highlighted a 20.2% incidence of symptomatic RP postchemoradiotherapy in 24,527 patients with LA-NSCLC. Notably, the analysis revealed a 33.6% occurrence of G2+ pneumonitis in patients who were treated with a combination of chemoradiotherapy and immunotherapy, indicating a higher risk of symptomatic RP than in patients receiving radiotherapy alone (21). Similarly, a comprehensive meta-analysis, which reviewed data from 836 patients NSCLC who received chemoradiotherapy, reported an overall incidence of symptomatic RP at 29.8% following chemoradiotherapy (22), which was lower than the 36.02% incidence of SP in this study. This suggests that incorporating immunotherapy elevates the risk of SP beyond grade 2.

### 4.2 SP-related factors analysis

In this study, the occurrence of SP was found to be independently linked to the ECOG PS score and T stage, which is consistent with existing research findings. The evaluation of tumor size through T staging shows that T1 tumors have a reduced risk of SP compared to T2 tumors (23), and tumors in stage I-II have an inferior risk of RP compared to those in stage III-IV (24). In addition to reflecting systemic immune status, immunological parameters offer insights into the immune microenvironment within tumor tissues. Studies indicate that these parameters correlate with lung cancer patient prognosis postradiotherapy and chemotherapy (25, 26). Our univariate analysis revealed a connection between the pre-RT PLR and the occurrence of SP, which is consistent with prior research. Shan et al. (27) indicated that the pre-treatment PLR is a predictor of RP occurrence in patients who are receiving stereotactic body radiation therapy (SBRT). Although dosimetric parameters did not show an association with SP in our study, recent research underscores their relevance to pulmonary toxicity in lung cancer patients undergoing radiotherapy with immunotherapy. Landman et al. (28) highlighted a significant relationship between pneumonia incidence in radioimmunoassay-treated lung cancer patients and higher bilateral lung V5, V20, and MLD. Additionally, Koffer et al. (29) reported that bilateral lung V20 and MLD can predict SP occurrence in lung cancer patients postcombined radioimmunotherapy. Dosimetric parameters were integrated into our model based on these studies.

### 4.3 Comparisons with other studies

Radiotherapy combined with immunotherapy offers a significant survival benefit for lung cancer patients; however, it also intensifies pulmonary toxicity. This study represents an earlier exploration into predicting pulmonary toxicity resulting from combined radioimmunotherapy. Lung cancer is a complex and heterogeneous disease, particularly in patients receiving combined radioimmunotherapy. These individuals often exhibit diverse baseline characteristics, such as multiple treatment cycles, underlying illnesses, and advanced age, which are common high-risk factors for pneumonia onset and vary widely across patient groups. Individual patient differences, and treatment sensitivities of tumors, clinical data, immunological factors, and dosimetric parameters derived from CT images are integrated to provide comprehensive and personalized insights recognizing the intrinsic heterogeneity and enhancing the accuracy of SP prediction. Krafft et al. (14) created a model to predict RP using pretreatment whole-lung radiomic features combined with clinical and dosimetric data, achieving an AUC of 0.68, which was consistent with our findings. Meanwhile, radiomics has made significant advancements in the diagnosis and prognosis of lung cancer. Lung cancer is one of the most prevalent and deadly malignancies globally, with a relatively low overall five-year survival rate. Therefore, early diagnosis and prognosis are crucial for improving patient survival rates. In recent years, the widespread application of artificial intelligence (AI) technologies in the field of lung cancer, particularly in natural language processing, machine learning, deep learning, and reinforcement learning, has significantly enhanced the accuracy of early diagnosis and prognostic predictions (30). Furthermore, the application of AI has propelled cancer prediction performance to new heights, not only improving diagnostic accuracy but also presenting new opportunities and challenges for clinical implementation (31). In addition, Qiu et al. (32) distinguished between CIP and RP based on pre-treatment CT images, revealing that clinical or radiological parameters such as bilateral lesions (p < 0.001) and sharp margins (p = 0.001) were significantly associated with the identification of CIP and RP. In the validation cohort, the AUC values reached 0.901 and 0.874, respectively. Yang et al. (33) explored the ability of CT radiomics features to predict the EGFR mutation status. The results showed that the AUC values of the unenhanced, arterial and venous phases in the EGFR mutation status training group were 0.6713, 0.8194 and 0.8464, respectively. Wen et al. (34) compared the predictive performance of pretreatment CT-based radiomics signatures and clinicopathological and CT morphological factors for PD-L1 expression level and tumor mutation burden (TMB) status. Radiomics signatures showed good performance for predicting PD-L1 and TMB with AUCs of 0.730 and 0.759, respectively. Predictive models that combined radiomics signatures with clinical and morphological factors dramatically improved the predictive efficacy for PD-L1 (AUC = 0.839) and TMB (AUC = 0.818). Radiomics can extract numerous features but may overlook subtle high-order characteristics. Hence, a DL approach is applied in this study based on the ResNet34 architecture for deep image feature extraction. Unlike manual feature extraction, DL eliminates the need for contouring, reducing variability among different segmentations and enhancing efficiency. Additionally, DL provides detailed information specific to each task within the hidden layers of the neural network. This amplifies the performance of the model by a significant margin. The multidimensional deep network model developed in this study outperforms several other models, demonstrating that integrating machine learning and clinical data can enrich image features with additional information, markedly enhancing predictive accuracy. Moreover, this study aims to create a decision-support tool for clinical use, emphasizing that optimal models often require the combination of multiple factors to enhance decision-making capabilities (12, 35). Consequently, the results affirm that models combining multiple indicators outperform single models. PCA is used to diminish the complexity of image features that have been extracted. This method allows the conversion of high-dimensional data into lower-dimensional form, ensuring that essential information is retained throughout the process. By selecting representative principal components, data redundancy is minimized, data analysis and visualization processes are simplified, and model performance is enhanced.

The combination of radiotherapy and immunotherapy has become the standard treatment for patients with locally advanced, unresectable NSCLC. While this combination enhances anti-tumor efficacy, it also increases the risk of pulmonary complications, particularly SP. The clinical significance of our model lies in its ability to identify independent risk factors, such as ECOG and T stage, which underscores the model’s clinical applicability. These factors are readily available in clinical practice, allowing for the model to be integrated into the routine assessment of lung cancer patients prior to initiating treatment. Moreover, our model relies on pre-treatment CT images, which means it can be easily implemented in clinical settings without the need for additional invasive procedures or extensive follow-up imaging. This convenience enhances its feasibility as a routine assessment tool. The ability to accurately predict SP is crucial for patient management. Our study presents a robust deep imaging radiomics model that serves as a decision-making tool, capable of precisely forecasting the occurrence of SP in lung cancer patients undergoing radiotherapy and immunotherapy. By facilitating the early identification of patients at high risk for SP, the model guides clinicians in proactive monitoring and intervention, enabling the selection of appropriate treatment strategies tailored to individual patient profiles. Consequently, treatment plans can be adjusted accordingly, mitigating the severity of pneumonitis and improving the safety of therapeutic regimens for this vulnerable population.

### 4.4 Limitations of the study

There are limitations to this research. First, being retrospective, it depends on data from solely one institution and lacks validation from external data; therefore, additional research is necessary to evaluate the model’s applicability. Second, the sample size was limited, underscoring the necessity for more extensive samples in forthcoming studies to improve model precision. Third, in addition to radiomic features, the present study incorporated only clinical, immunological, and dosimetric parameters, omitting biological factors. Studies show that increased baseline levels of GM-CSF and sIL-6R are associated with the development of pneumonitis in lung cancer patients receiving radiochemotherapy and immunotherapy (36), variables not included in this study. Genomic or proteomic data can elucidate pulmonary toxic response mechanisms, suggesting that integrating radiomic features with such data can enhance predictive capabilities. Thus, future research should explore new data types and incorporate additional information to refine the prediction accuracy. Moreover, this study utilized only dose and volume histogram (DVH) parameters to represent the two-dimensional dose distribution within the target body and failed to capture spatial dose distribution characteristics. RP primarily manifests in the locally irradiated lung region. Multiregion-based radiomics can depict the three-dimensional spatial dose distribution and can extract radiomic features from multiple regions, including high-, medium-, and low-dose areas and specific-sized 3D rings formed around the tumor, capturing predictive indicators for the occurrence of pulmonary toxicity better. While this study extracted radiomics features from normal lung tissue and combined them with two-dimensional dose parameters to construct a model, future investigations can incorporate multi-regional three-dimensional omics analysis for enhanced predictive outcomes.

## 5 Conclusion

To summarize, the research findings indicate that an extensive deep neural network model utilizing deep image features and radiomics features from preradiotherapy CT images, along with clinical data, is highly effective in forecasting the development of SP in lung cancer patients undergoing radiotherapy in conjunction with immunotherapy. Subsequent studies should prioritize expanding the sample size and integrating data from multiple centers for external validation to bolster the reliability and precision of the model, thereby enabling healthcare providers to make well-informed and accurate clinical judgments.

## Data Availability

The original contributions presented in the study are included in the article/**Supplementary Material**. Further inquiries can be directed to the corresponding author.
