# Supplementary figures and images for: Multimodal data deep learning method for predicting symptomatic pneumonitis caused by lung cancer radiotherapy combined with immunotherapy
Source: Front Immunol. 2025 Jan 8;15:1492399. doi: 10.3389/fimmu.2024.1492399 (PMC11751032; doi:10.3389/fimmu.2024.1492399)

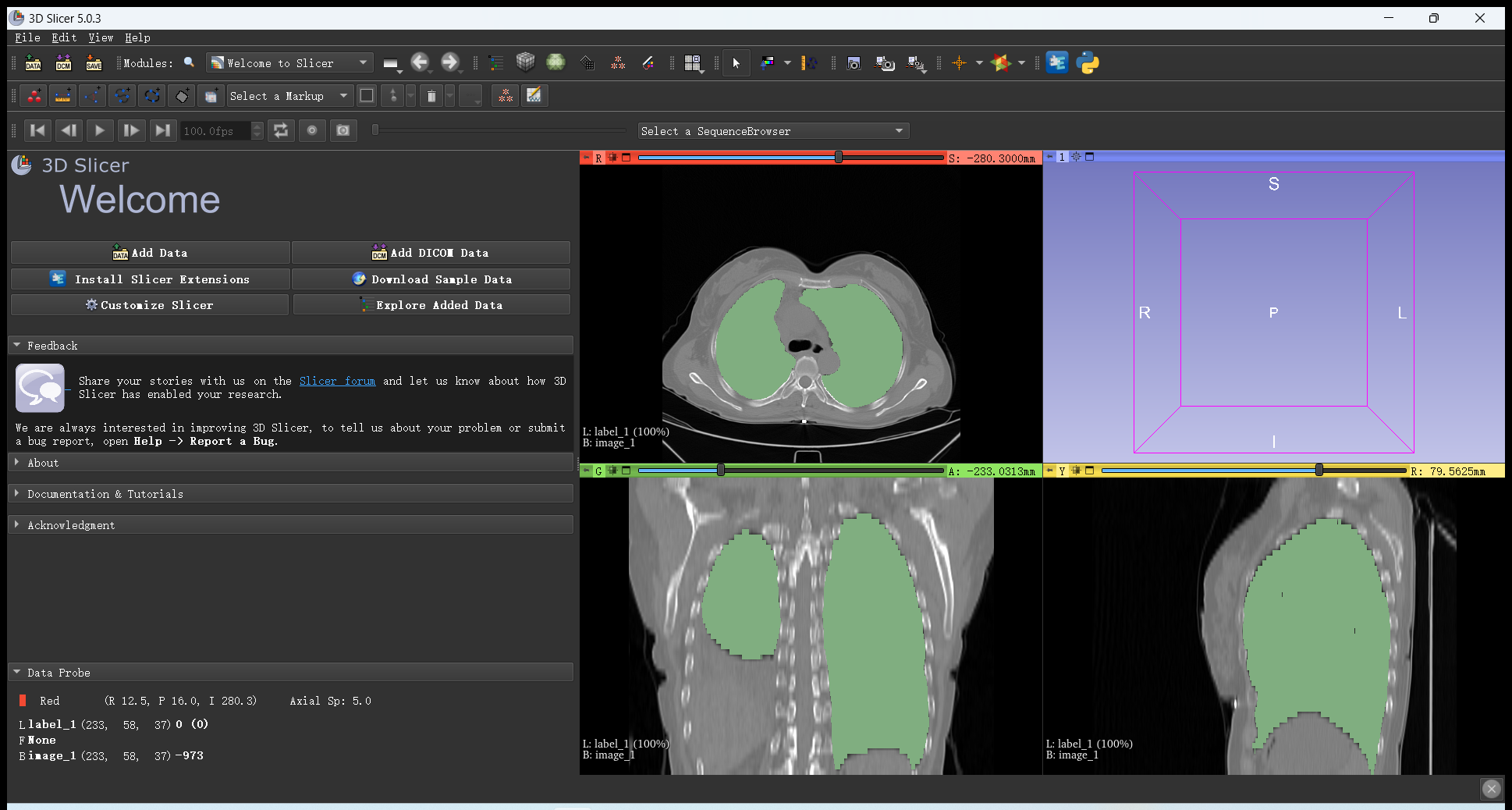

Supplement: Supplementary file 1 [file Image1.png]

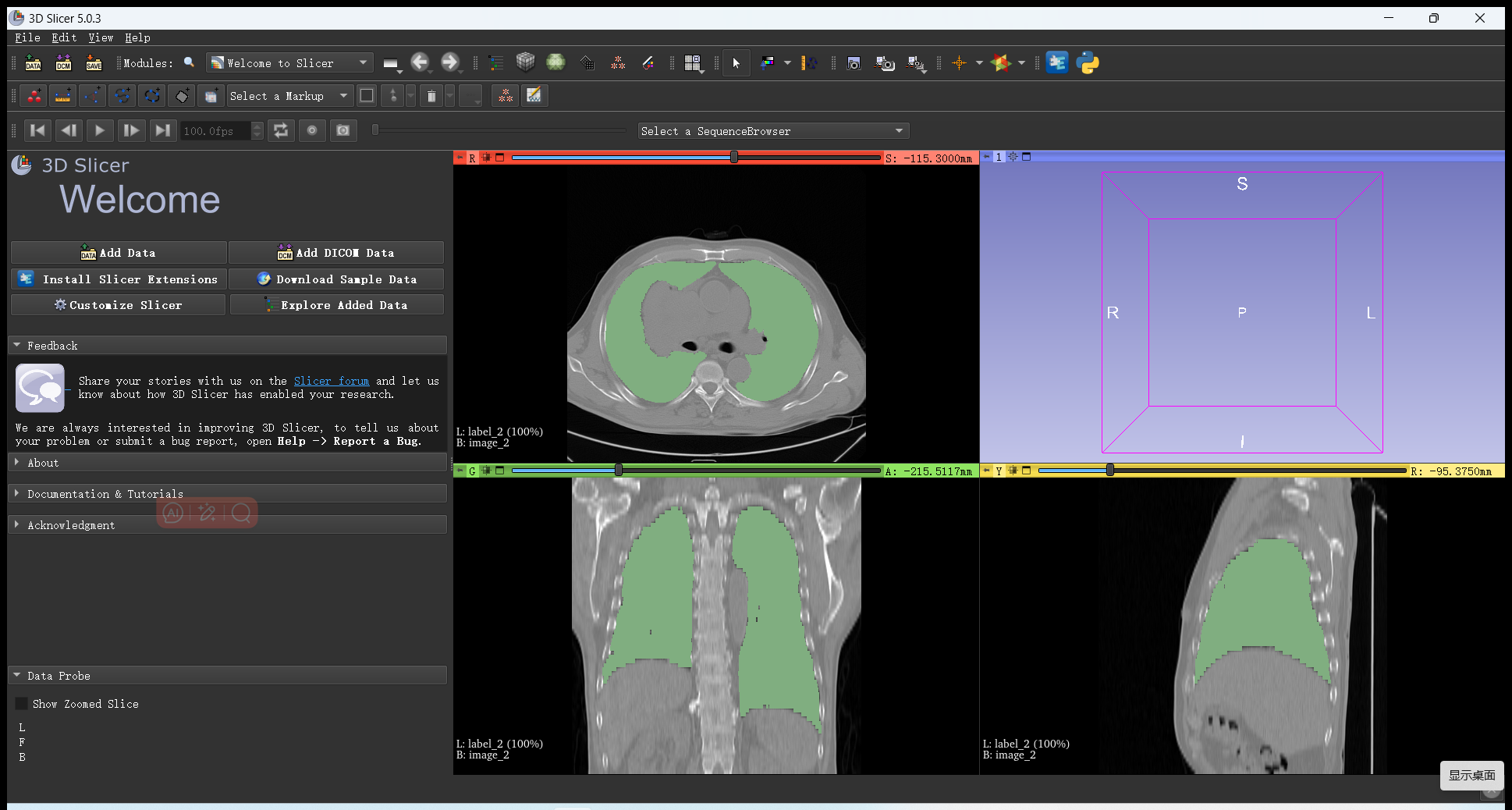

Supplement: Supplementary file 2 [file Image2.png]

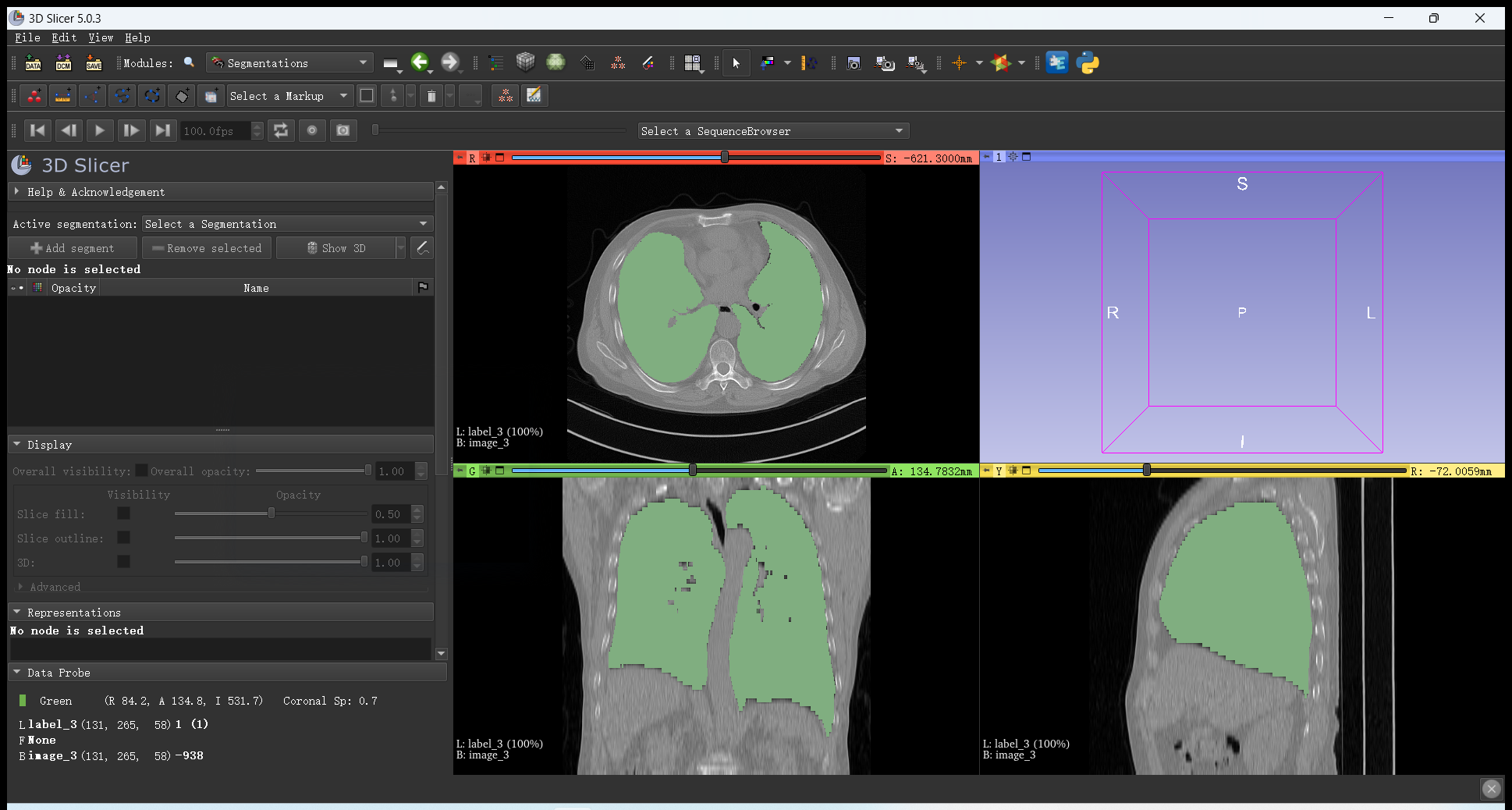

Supplement: Supplementary file 3 [file Image3.png]
