# Supplementary material for: Multimodal data deep learning method for predicting symptomatic pneumonitis caused by lung cancer radiotherapy combined with immunotherapy
Source: Front Immunol. 2025 Jan 8;15:1492399. doi: 10.3389/fimmu.2024.1492399 (PMC11751032; doi:10.3389/fimmu.2024.1492399)
Supplement: Supplementary file 4 [file Table1.docx]

TABLE 1 The structural parameters of ResNet34

| Index | Layer Name | Parameters |
| --- | --- | --- |
| 1 | Convolutional Layer | 7$\times$7,64,stride=2 |
| 2 | Pooling Layer | 3$\times$3 max pool, stride=2 |
| 3 | Convolutional Layers | 3$\times\left\{ \begin{aligned} 3\times3, 64 \\ 3\times3, 64 \end{aligned} \right.$ |
| 4 | Convolutional Layers | 4$\times\left\{ \begin{aligned} 3\times3, 128 \\ 3\times3, 128 \end{aligned} \right.$ |
| 5 | Convolutional Layers | 6$\times\left\{ \begin{aligned} 3\times3, 256 \\ 3\times3, 256 \end{aligned} \right.$ |
| 6 | Convolutional Layers | 3$\times\left\{ \begin{aligned} 3\times3, 512 \\ 3\times3, 512 \end{aligned} \right.$ |
| 7 | Pooling Layer | Average pool, 512-D output |

TABLE 2 Baseline characteristics

| Characteristics | Non-symptomatic pneumonia(n=167) | Symptomatic pneumonia(n=94) | All(n=261) | P value |
| --- | --- | --- | --- | --- |
| Age,(y) |  |  |  | 0.698 |
| <61 | 77(46.1) | 41(43.6) | 118(45.2) |  |
| ≥61 | 90(53.9) | 53(56.4) | 143(54.8) |  |
| Gender |  |  |  | 0.503 |
| Male | 131(78.4) | 77(81.9) | 208(79.7) |  |
| Female | 36(21.6) | 17(18.1) | 53(20.3) |  |
| ECOG PS |  |  |  | 0.011 |
| 0-1 | 161(96.4) | 83(88.3) | 244(93.5) |  |
| ≥2 | 6(3.6) | 11(11.7) | 17(6.5) |  |
| Smoker History |  |  |  | 0.793 |
| No | 81(48.5) | 44(46.8) | 125(47.9) |  |
| Yes | 86(51.5) | 50(53.2) | 136(52.1) |  |
| Smoking Index |  |  |  | 0.273 |
| <400 | 97(58.1) | 48(51.1) | 145(55.6) |  |
| ≥400 | 70(41.9) | 46(48.9) | 116(44.4) |  |
| Tumor Histology |  |  |  | 0.269 |
| Squamous Cell Carcinoma | 76(45.5) | 48(51.1) | 124(47.5) |  |
| Adenocarcinoma | 34(20.4) | 18(19.1) | 52(19.9) |  |
| Small Cell Lung Cancer | 55(32.9) | 24(25.5) | 79(30.3) |  |
| Other | 2(1.2) | 4(4.3) | 6(2.3) |  |
| T Stage |  |  |  | 0.000 |
| 1 | 63(37.7) | 16(17.0) | 79(30.3) |  |
| 2 | 54(32.3) | 34(36.2) | 88(33.7) |  |
| 3 | 34(20.4) | 30(31.9) | 64(24.5) |  |
| 4 | 16(9.6) | 14(14.9) | 30(11.5) |  |
| N Stage |  |  |  | 0.779 |
| 0 | 32(19.2) | 11(11.7) | 43(16.5) |  |
| 1 | 4(2.4) | 4(4.3) | 8(3.1) |  |
| 2 | 91(54.5) | 60(63.8) | 151(57.9) |  |
| 3 | 40(24.0) | 19(20.2) | 59(22.6) |  |
| M Stage |  |  |  | 0.937 |
| 0 | 104(62.3) | 59(62.8) | 163(62.5) |  |
| 1 | 63(37.7) | 35(37.2) | 98(37.5) |  |
| Radiotherapy Dose |  |  |  | 0.236 |
| <60Gy | 37(22.2) | 27(28.7) | 64(24.5) |  |
| ≥60Gy | 130(77.8) | 67(71.3) | 197(75.5) |  |
| Radiotherapy Fractions |  |  |  | 0.356 |
| <30f | 36(21.6) | 25(26.6) | 61(23.4) |  |
| ≥30f | 131(78.4) | 69(73.4) | 200(76.6) |  |
| Chemotherapy Cycles |  |  |  | 0.596 |
| <4 | 31(18.6) | 20(21.3) | 51(19.5) |  |
| ≥4 | 136(81.4) | 74(78.7) | 210(80.5) |  |
| Cycles of Immunotherapy | 4.00(2.00-6.00) | 4.00(2.00-5.00) | 4.00(2.00-6.00) | 0.474 |
| Type of immunotherapy |  |  |  | 0.158 |
| Sintilimab | 19(11.4) | 7(7.4) | 26(10.0) |  |
| Camrelizumab | 52(31.1) | 22(23.4) | 74(28.4) |  |
| Pembrolizumab | 11(6.6) | 11(11.7) | 22(8.4) |  |
| Toripalimab | 7(4.2) | 10(10.6) | 17(6.5) |  |
| Atezolizumab | 11(6.6) | 4(4.3) | 15(5.7) |  |
| Tislelizumab | 23(13.8) | 19(20.2) | 42(16.1) |  |
| Durvalumab | 19(11.4) | 6(6.4) | 25(9.6) |  |
| Serplulimab | 6(3.6) | 3(3.2) | 9(3.4) |  |
| Sugemalimab | 2(1.2) | 0(0.0) | 2(0.8) |  |
| Combination immunotherapy drugs | 17(10.2) | 12(12.8) | 29(11.1) |  |
| NLR1 | 2.40(1.70-3.50) | 2.70(1.80-4.35) | 2.50(1.70-3.75) | 0.070 |
| NLR2 | 4.30(2.90-7.50) | 4.55(2.98-7.60) | 4.50(2.95-7.55) | 0.607 |
| NLR3 | 4.20(2.60-6.60) | 4.70(3.10-7.35) | 4.40(2.90-6.95) | 0.214 |
| PLR1 | 122.10(97.90-175.60) | 146.10(98.98-212.68) | 126.70(97.90-186.45) | 0.078 |
| PLR2 | 219.30(163.00-310.90) | 224.85(159.65-327.30) | 219.30(160.65-315.50) | 0.795 |
| PLR3 | 239.80(157.30-357.80) | 253.60(147.60-416.45) | 241.80(156.25-371.00) | 0.667 |
| LMR1 | 3.70(2.60-5.00) | 3.05(2.20-4.30) | 3.40(2.40-4.60) | 0.016 |
| LMR2 | 1.70(1.20-2.30) | 1.70(1.10-2.23) | 1.70(1.10-2.30) | 0.550 |
| LMR3 | 1.90(1.20-2.70) | 1.60(1.20-2.60) | 1.80(1.20-2.60) | 0.504 |
| SII1 ( x 10^9^/L) | 486.40(331.30-850.70) | 608.65(372.10-1013.95) | 519.70(350.15-912.90) | 0.090 |
| SII2 ( x 10^9^/L) | 806.20(531.70-1349.40) | 870.10(540.60-1459.45) | 826.10(539.75-1371.95) | 0.949 |
| SII3 ( x 10^9^/L) | 898.20(524.00-1428.60) | 944.85(537.85-1745.35) | 940.50(533.65-1550.55) | 0.417 |
| V_5_ | 40.94(33.37-46.00) | 41.48(33.99-49.04) | 41.00(33.69-46.75) | 0.281 |
| V_20_ | 22.17(18.02-25.00) | 22.05(18.69-26.00) | 22.10(18.11-25.06) | 0.257 |
| V_30_ | 16.35(12.78-19.21) | 16.43(12.98-20.05) | 16.38(12.85-19.70) | 0.312 |
| MLD (Gy) | 12.07(10.06-13.88) | 12.15(10.21-14.93) | 12.13(10.11-14.11) | 0.359 |

Note: Combination immunotherapy drugs: Using more than two immunological drugs in combination; NLR1: NLR before radiotherapy; NLR2: NLR in radiotherapy; NLR3: NLR after radiotherapy; PLR1: PLR before radiotherapy; PLR2: PLR in radiotherapy; PLR3: PLR after radiotherapy; LMR1: LMR before radiotherapy; LMR2: LMR in radiotherapy; LMR3: LMR after radiotherapy; SII1: SII before radiotherapy; SII2: SII in radiotherapy; SII3: SII after radiotherapy; V5: Lung volume at least 5 Gy irradiated; V20: Lung volume at least 20 Gy irradiated; V30: Lung volume at least 30 Gy irradiated;

TABLE 3 Results of both univariate and multivariate analyses on SP

|  | Univariate analysis | |  | Multivariate analysis | |
| --- | --- | --- | --- | --- | --- |
|  | HR（95% CI） | P value |  | HR（95% CI） | P value |
| Age | 1.022（0.990-1.055） | 0.185 |  |  |  |
| Gender | 0.803（0.423-1.526） | 0.504 |  |  |  |
| ECOG PS | 3.556（1.270-9.955） | **0.016** |  | 3.322（1.148-9.615） | **0.027** |
| Smoker History | 0.934（0.563-1.550） | 0.793 |  |  |  |
| Smoking Index | 0.753（0.453-1.251） | 0.274 |  |  |  |
| Tumor Pathology | 0.923（0.701-1.215） | 0.566 |  |  |  |
| T-Stage | 1.558（1.198-2.026） | **0.001** |  | 1.501（1.147-1.965） | **0.003** |
| N-Stage | 1.111（0.847-1.457） | 0.446 |  |  |  |
| M-Stage | 0.979（0.581-1.651） | 0.937 |  |  |  |
| Radiotherapy Dose | 1.416（0.795-2.521） | 0.238 |  |  |  |
| Radiotherapy Fractions | 1.318（0.733-2.373） | 0.357 |  |  |  |
| Chemotherapy Cycles | 1.186（0.632-2.225） | 0.596 |  |  |  |
| Cycles of Immunotherapy | 0.971（0.909-1.037） | 0.374 |  |  |  |
| Type of immunotherapy | 1.032（0.945-1.127） | 0.485 |  |  |  |
| NLR1 | 1.071（0.987-1.162） | 0.100 |  |  |  |
| NLR2 | 1.010（0.952-1.072） | 0.741 |  |  |  |
| NLR3 | 1.018（0.988-1.049） | 0.238 |  |  |  |
| PLR1 | 1.003（1.000-1.006） | **0.028** |  | 1.003（1.000-1.006） | 0.059 |
| PLR2 | 1.000（0.998-1.002） | 0.924 |  |  |  |
| PLR3 | 1.000（0.999-1.002） | 0.694 |  |  |  |
| LMR1 | 0.920（0.837-1.012） | 0.086 |  |  |  |
| LMR2 | 1.015（0.834-1.234） | 0.884 |  |  |  |
| LMR3 | 0.988（0.868-1.125） | 0.856 |  |  |  |
| SII1 ( x 10^9^/L) | 1.000（1.000-1.001） | 0.124 |  |  |  |
| SII2 ( x 10^9^/L) | 1.000（1.000-1.000） | 0.653 |  |  |  |
| SII3 ( x 10^9^/L) | 1.000（1.000-1.000） | 0.170 |  |  |  |
| V_5_ | 1.019（0.994-1.045） | 0.128 |  |  |  |
| V_20_ | 1.038（0.992-1.087） | 0.106 |  |  |  |
| V_30_ | 1.040（0.989-1.094） | 0.123 |  |  |  |
| MLD (Gy) | 1.059（0.982-1.143） | 0.139 |  |  |  |

Note: HR: Hazard ratio; CI: Confidence interval; NLR1: NLR before radiotherapy; NLR2: NLR in radiotherapy; NLR3: NLR after radiotherapy; PLR1: PLR before radiotherapy; PLR2: PLR in radiotherapy; PLR3: PLR after radiotherapy; LMR1: LMR before radiotherapy; LMR2: LMR in radiotherapy; LMR3: LMR after radiotherapy; SII1: SII before radiotherapy; SII2: SII in radiotherapy; SII3: SII after radiotherapy; V5: Lung volume at least 5 Gy irradiated; V20: Lung volume at least 20 Gy irradiated; V30: Lung volume at least 30 Gy irradiated;

TABLE 4 Performances evaluation sheet for all models

|  | AUC(95%CI) | Sensitivity | Specificity | Accuracy | Precision | F1 score | MCC | Kappa | P value |
| --- | --- | --- | --- | --- | --- | --- | --- | --- | --- |
| RF_Traditional Omics | 0.576(0.523-0.628) | 0.818 | 0.316 | 0.635 | 0.675 | 0.740 | 0.153 | 0.145 | - |
| RF_Clinical Information | 0.525(0.479-0.572) | 0.818 | 0.211 | 0.596 | 0.643 | 0.720 | 0.035 | 0.032 | - |
| RF_Traditional Omics_Clinical Information | 0.611(0.566-0.652) | 0.788 | 0.368 | 0.635 | 0.684 | 0.732 | 0.170 | 0.165 | - |
| DNN_Traditional Omics | 0.811(0.786-0.832) | 0.727 | 0.737 | 0.731 | 0.828 | 0.774 | 0.450 | 0.444 | <0.001^a^ |
| DNN_Clinical Information | 0.711(0.682-0.753) | 0.647 | 0.667 | 0.654 | 0.786 | 0.710 | 0.299 | 0.291 | <0.001^b^ |
| DNN_Traditional Omics_Clinical Information | 0.872(0.845-0.896) | 0.697 | 0.842 | 0.750 | 0.885 | 0.800 | 0.519 | 0.500 | <0.001^c^ |
| Our Model | 0.922(0.902-0.945) | 0.879 | 0.789 | 0.846 | 0.879 | 0.879 | 0.668 | 0.668 | <0.001^d^ |

Note: a: The AUC of a DNN model using traditional radiomic features versus the AUC of an RF model using traditional radiomic features by Delong test, P < 0.001. b: The AUC of a DNN model using clinical data versus the AUC of an RF model using clinicaldata by Delong test, P < 0.001. c: The AUC of a DNN model integrating conventional radiomic features with clinical data versus the AUC of an RF model integrating conventional radiomic features with clinical data by Delong test, P < 0.001. d: The AUC of our model versus the AUC of DNN models by Delong test, P < 0.001.
